# Scaling medical device regulatory science using large language models

**DOI:** 10.1038/s41746-026-02353-7

**Published:** 2026-02-05

**Authors:** Hanyang Li, Xiao He, Adarsh Subbaswamy, Patrick Vossler, Alexej Gossmann, Karandeep Singh, Jean Feng

**Affiliations:** 1 Unaffiliated, San Francisco, California, CA USA; 2 University of Maryland, Baltimore, MD USA; 3 University of California, San Francisco, CA USA; 4 Unaffiliated, Annandale, VA USA; 5 University of California, San Diego, CA USA

**Keywords:** Computational biology and bioinformatics, Engineering, Health care, Mathematics and computing, Medical research

## Abstract

Advances in artificial intelligence (AI) and machine learning (ML) have led to a surge in AI/ML-enabled medical devices, posing new challenges for regulators because best practices for developing, testing, and monitoring these devices are still emerging. Consequently, there is a critical need for up-to-date data analyses of the regulatory landscape to inform policy-making. However, such analyses have historically relied upon manual annotation efforts because regulatory documents are unstructured, complex, multi-modal, and filled with jargon. Efforts to automate annotation using simple natural language processing methods have achieved limited success, as they lack the reasoning needed to interpret regulatory materials. Recent progress in large language models (LLMs) presents an unprecedented opportunity to unlock information embedded in regulatory documents. This work conducts the first wide-ranging validation study of LLMs for scaling data analyses in the field of medical device regulatory science. Evaluating LLM outputs using expert manual annotations and “LLM-as-a-judge,” we find that LLMs can accurately extract attributes spanning pre- and post-market settings, with accuracy rates often reaching 80% or higher. We then demonstrate how LLMs can scale up analyses in three applications: (1) monitoring device validation practices, (2) coding medical device reports, and (3) identifying potential risk factors for post-market adverse events.

## Introduction

The regulatory landscape for artificial intelligence (AI) and machine learning (ML)-enabled medical devices is rapidly evolving as technological advances in AI are being integrated into medical products. In the United States, the number of FDA-authorized AI/ML-enabled medical devices has grown from fewer than 100 in 2018 to over 1247 by mid-2025^1^. To address the unique regulatory challenges posed by these devices, particularly frequent updates to the underlying models post-deployment, the US Food and Drug Administration (FDA) published a 2019 discussion paper^2^ proposing a regulatory framework for modifications to AI/ML-based software as a medical device (SaMD). This was followed by the AI/ML-Based SaMD Action Plan in 2021^3^, final guidance on Predetermined Change Control Plans (PCCPs) in 2024^4^ to let manufacturers pre-specify updates to their AI models without needing new regulatory submissions, and most recently, a draft guidance on lifecycle management and marketing submissions for AI-enabled device software functions in 2025^5^. These developments have resulted in a growing number of studies analyzing various regulatory documents to understand the types of devices and regulatory approval/clearance patterns that have emerged in response to the rise of AI/ML in healthcare^6–12^ These studies from regulatory science have highlighted the need for timely, data-driven insights into how policies and technological innovation interact, which can inform future regulatory strategies, product development choices, and industry best practices.

Nevertheless, conducting data analysis in medical device regulatory science in a timely fashion is challenging, as the process is currently expensive and time-consuming. Prior works have relied heavily on manual extraction of information from regulatory documents, which often lack standardization, can be multi-modal, and make heavy use of jargon. Furthermore, the manual nature of these analyses makes them difficult to repeat and quick to become out of date, especially given the rapid growth in the number of AI/ML-enabled medical devices being authorized by the FDA^13,14^, While recent works have incorporated Natural Language Processing (NLP), the focus has been on exact pattern matching, which is effective for well-structured, regular pieces of information (e.g., predicate device numbers)^9,15–17^, but less so for more complex concepts. Consider three examples:

1. **Surveying device validation practices**: To obtain FDA authorization for a device, manufacturers typically present results from one or more validation studies of a medical device to establish its safety and effectiveness for the intended use^18,19^, After receiving FDA authorization, manufacturers often prepare and submit a document summarizing the decision process (i.e., “decision summary”) to be publicly published on the FDA website. To examine the types of studies used to authorize AI/ML-based medical devices, Wu et al. 2021 manually reviewed 130 decision summaries that were available at the time^6^. As of the latest data release in July 2025, there are now 1247 such devices.
2. **Coding of medical device reports**: To assess the quality of post-market surveillance data, prior works have manually reviewed medical device reports (MDRs), which are reports submitted to the FDA to document adverse events, malfunctions, or other issues potentially associated with a medical device. These reports are publicly available in the FDA’s Manufacturer and User Facility Device Experience (MAUDE) database. Manual analyses of MAUDE have revealed that MDR event types contain systematic missingness and misclassifications in the vendor-reported data^20,21^, A recent study reviewed event type codes for a subset of the 943 MDRs available at the time^7^. As of August 2025, that number has grown to 1852 MDRs for AI/ML-enabled medical devices alone. Although recent works have suggested using pattern-matching methods to identify MDRs that have been potentially misclassified^17^, determining the true label requires complex reasoning about whether the device has likely caused or contributed to death and/or injury^22^.
3. **Identifying premarket factors associated with safety risks**: Medical devices in the United States may be cleared or approved through one of several FDA regulatory pathways, including the 510(k) premarket notification, the de novo classification, and the premarket approval (PMA) process. Among these, the 510(k) pathway is by far the most commonly used, accounting for over 90% of AI/ML-enabled device clearances and ~3000 clearances annually^23,24^, This pathway requires manufacturers to demonstrate that their device is substantially equivalent (SE) to a legally marketed predicate device in terms of safety and efficacy. To identify potential factors during the 510(k) clearance process that may be associated with increased post-market safety risks, prior works have manually reviewed individual devices and traced their predicate device ancestry^25,26^, While recent works have used pattern-matching methods to extract predicate device numbers^15,27^, such approaches have difficulty extracting complex risk factors (e.g., the type of device validation study). As such, there is a need for methods that can generate generalizable, rigorous evidence by analyzing all FDA-cleared devices and their predicate lineage, and rigorous statistical analyses to link premarket characteristics to post-market patient outcomes.

With the latest advancements in AI, particularly large language models (LLMs), there is a unique opportunity to substantially accelerate medical device regulatory science by automating the extraction of structured data from documents that were previously difficult to analyze. Indeed, AI/LLMs have already accelerated operational and clinical areas in other areas of regulatory science, including hospital quality measurement, drug development, and clinical trial matching^28–30^ Although the use of LLMs specifically for regulatory lifecycle analysis of medical devices has been underexplored^31^, LLMs are particularly well-suited for research in this area: regulatory submission documents and guidances are often long, the publicly available information is unstructured, and regulatory pathways are interconnected in complex ways. At the same time, there is a need for caution: LLMs are known to hallucinate, propagate bias, and require careful validation^32,33^, Consequently, there is a need to comprehensively evaluate LLMs across a broad range of data extraction tasks to determine if they can enable scalable, reproducible, and up-to-date regulatory science of medical devices.

To this end, we present a general LLM pipeline that integrates public FDA decision summaries, the MAUDE database, and device recall histories to analyze 1247 FDA-authorized AI/ML-enabled medical devices and 1852 MDRs, the largest and most comprehensive such analysis to date. After validating the LLM extractions, we use it to revisit and scale up analyses for the three aforementioned questions: surveying device clearance practices, coding medical device reports, and identifying premarket factors linked to higher safety risk.

## Results

The dataset is composed of all AI/ML-enabled devices approved or cleared by the FDA (*n* = 1247) and their MDRs (*n* = 1852) up to July 2025, retrieved through the FDA’s APIs. After removing devices and AEs with missing decision summary PDFs or MDR text, this resulted in a final list of 1227 summaries and 1841 MDRs. Device decision summaries and MAUDE reports consist primarily of unstructured data, where information is often presented in a highly variable manner with domain- or industry-specific terminology. Many documents lack structural consistency, mixing narrative descriptions with semi-formatted tables or images. For instance, clearance of a device often requires multiple validation studies, which are discussed with varying levels of detail; some summaries provide only a few sentences while others discuss test results over multiple pages. As such, extracting precise information—such as differentiating between primary and secondary predicate devices, or isolating causes of adverse events—requires not just comprehension but inference, reasoning, and contextual disambiguation. A summary of the data characteristics are provided in Table 5.

**Fig. 1:**
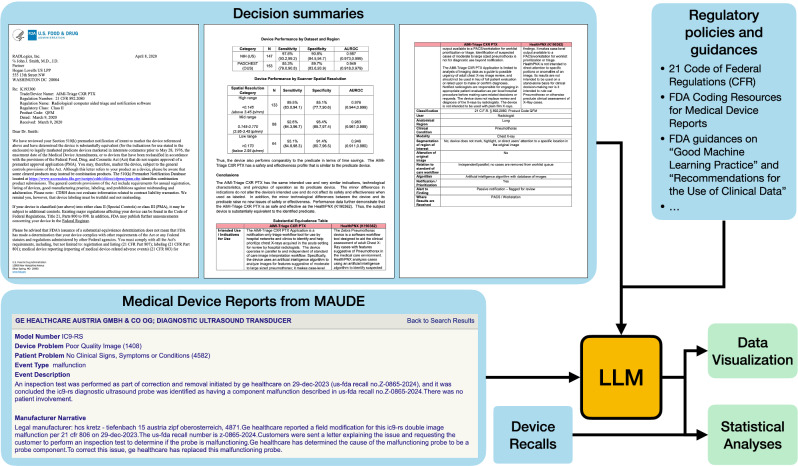
Large language model (LLM) pipeline for analyzing regulatory documents for AI/ML-enabled medical devices. Blue boxes indicate the inputs and preprocessing steps, while green boxes indicate downstream analyses that ingest outputs from the LLM pipeline.

The LLM pipeline (Fig. 1) for extracting structured information from unstructured regulatory documents relies on a combination of careful data preprocessing and GPT-4.1 from OpenAI. For FDA decision summaries, text was extracted from the PDFs using PDF-to-text extraction methods and Optical Character Recognition (OCR) as a fallback, to ensure complete and accurate text capture. The extracted text is then routed through a series of modular subtasks to improve interpretability and reduce error propagation. Critically, LLMs were able to adapt to the diverse formats, jargon, and reporting styles found across FDA summaries and MAUDE reports. This flexibility allowed us to generalize across thousands of submissions without designing task-specific parsers for each document. To improve extraction accuracy, the LLM prompts incorporated detailed instructions from regulatory documents and in-context learning (ICL) examples. Details of the LLM pipeline are provided in the Methods section, including pipeline outputs in Table 6. Code is publicly available at https://github.com/jjfenglab/LLM-FDA-device.

Below, we present three case studies to illustrate how the LLM pipeline can scale up analyses of regulatory data. Each case study begins with evaluating the performance of the LLM pipeline on an annotated subset of the data and then presents results on LLM extractions across all available documents. Because the underlying regulatory data can be incomplete, findings reflect not only current regulatory practices but also the current level of transparency in regulatory documents. Finally, as a reference, we include performance comparisons between the LLM pipeline and pattern-matching methods in Table 7.

### Case study 1: Surveying device validation practices

We begin with revisiting ref. ^6^, which manually reviewed clinical validation studies described in public decision summaries for 130 FDA-approved AI/ML medical devices. Here, we consider two main attributes extracted in this prior work: whether the study was conducted prospectively and how many sites were involved. Comparing the LLM pipeline extractions against the human-annotated labels, the LLM pipeline had high agreement with the human annotations, with agreement rates of over 82% (Table 1). Given the small number of prospective studies and multi-site studies in ref. ^6^, we also sampled an additional set of 78 medical devices for human annotation, stratified by whether LLM labeled the study as being prospective, multi-site, and/or neither (see Methods). Within this targeted dataset, we find that the LLM pipeline achieves similar performance. For reference, interrater agreement rates on this dataset were 91% (79%, 100%).

**Table 1:** Agreement rates with human annotations for device validation studies conducted for FDA clearance/approval

Attribute	Compared to annotations from ref. ^5^ (*n* = 130)			Compared to annotations in this paper (*n* = 78)		
Continuous	Accuracy	MAE		Accuracy	MAE	
# of Sites	82% (76%, 89%)	0.42 (0.15, 0.69)		82% (74%, 91%)	0.58 (0.083, 1.1)	
**Binary**	Accuracy	TPR	1 – FPR	Accuracy	PPV	NPV
Multi-site	86% (80%, 92%)	90% (79%, 100%)	85% (78%, 92%)	90% (83%, 97%)	85% (74%, 95%)	97% (91%, 100%)
Prospective	97% (94%, 100%)	100%* (100%, 100%)	98% (95%, 100%)	90% (83%, 97%)	82% (68%, 96%)	94% (87%, 100%)

95% confidence intervals are shown in parentheses. Accuracy is defined as the frequency of an exact match.

*MAE* mean absolute error, *TPR* true positive rate, *FPR* false positive rate, *PPV* positive predictive value, *NPV* negative predictive value.

*The LLM technically disagreed with the prospective attribute annotation for K182513. However, upon inspection of this device, we agree with the LLM’s label, as the prospective testing conducted in K182513 was on contrived samples.

**Fig. 2:**
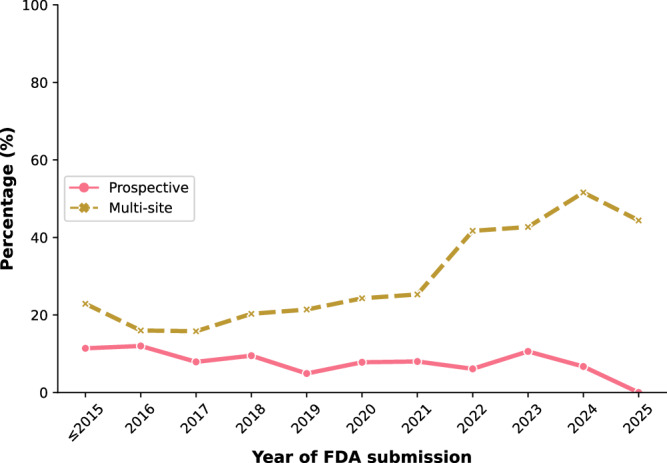
Trends in device validation practices. Temporal trends of decision summaries describing prospective validation (pink solid line) and multi-site validation (gold dashed line) of AI/ML-based medical devices cleared or approved by the FDA.

Using the LLM pipeline, we then extracted device validation practices described in the decision summaries for all FDA-authorized AI/ML medical devices spanning 1995 to 2025, to determine whether validation practices have changed since this initial analysis (Fig. 2). We observe that the rate of prospective evaluations has remained relatively consistent around 10% throughout the study period; this may reflect the fact that the FDA primarily requires prospective studies for certain classes of devices or for certain approval pathways. In contrast, there was a substantial increase in multi-site evaluations being mentioned, which rose from an initial rate of 20% to over 50%.

### Case study 2: Coding of medical device reports (MDRs)

Next, we revisit prior works that found MDRs can often be mis-coded^20,21,34^. Here, we focus on two classification schemes for MDRs: event type and device product problem (DPP). Event type describes whether the MDR reportable event describes an incident where the device caused or contributed to death (“death” type), caused or contributed to a serious injury (“injury” type), or whether it should be considered a malfunction (“malfunction”). DPP codes describe the device failures or problems observed during the event and should be chosen from an ontology of 487 IMDRF codes that vary in their granularity. Per FDA coding resources, vendors are instructed to “select the lowest level, most detailed code or codes that most accurately describe the device failures or problems observed during the event”^35^. There are multiple potential sources of bias and error when coding these two attributes, since the assigned code can vary based on the perspective of the coder^20^ and there is limited standardization of coding practices^21^, and the text descriptions provided for an MDR may be incomplete^22^.

To study whether LLMs can improve the alignment between codes assigned to MDRs and their provided descriptions, we used the LLM pipeline to re-annotate the event type and DPP labels for all MDRs, given the FDA’s instructions, but blinded to the original codes assigned by the manufacturer. To evaluate the LLM extractions, we randomly selected 89 MDRs where the LLM and vendor disagreed on some code and had four human annotators judge which assigned code(s) was more appropriate. As shown in Table 2, human annotators preferred the LLM-assigned codes 88% of the time for event types and 74% of the time for DPPs. For reference, the interrater agreement rates were 81% (64%, 98%) and 76% (61%, 91%) for Event Type and DPP, respectively. To complement the small human-annotated dataset, we employed the complementary approach of “LLM-as-a-judge” to scale up annotation for *all* MDRs at the expense of potential bias^36,37^. That is, we used an LLM to judge whether an LLM-extracted versus vendor-provided code is more appropriate; to mitigate self-preference bias, we used both GPT-4.1 and Claude Sonnet 4.5 as judges. The LLM judges preferred LLM-assigned codes over vendor-assigned ones at similar rates. This aligns with prior works, which have shown that LLM-as-a-judge can offer large-scale approximate validation^37^.

**Table 2:** Ranking results for LLM versus vendor assignments for event type and device product problem codes to medical device reports

	Judge	# total	# of LLM wins (% of non-ties)	# of vendor wins (% of non-ties)	# of ties
**Event type**	Human	90	40.3 (88%)	5.7 (12%)	44
	GPT-4.1	1841	102 (86%)	17 (14%)	1722
	Claude Sonnet 4.5	1841	80 (67%)	39 (33%)	1722
**Device product problem**	Human	90	60.8 (74%)	21.7 (26%)	7.5
	GPT-4.1	1841	1250 (80%)	303 (20%)	288
	Claude Sonnet 4.5	1841	1278 (83%)	263 (17%)	300

Only MDRs with non-empty vendor labels were included in this validation study.

**Fig. 3:**
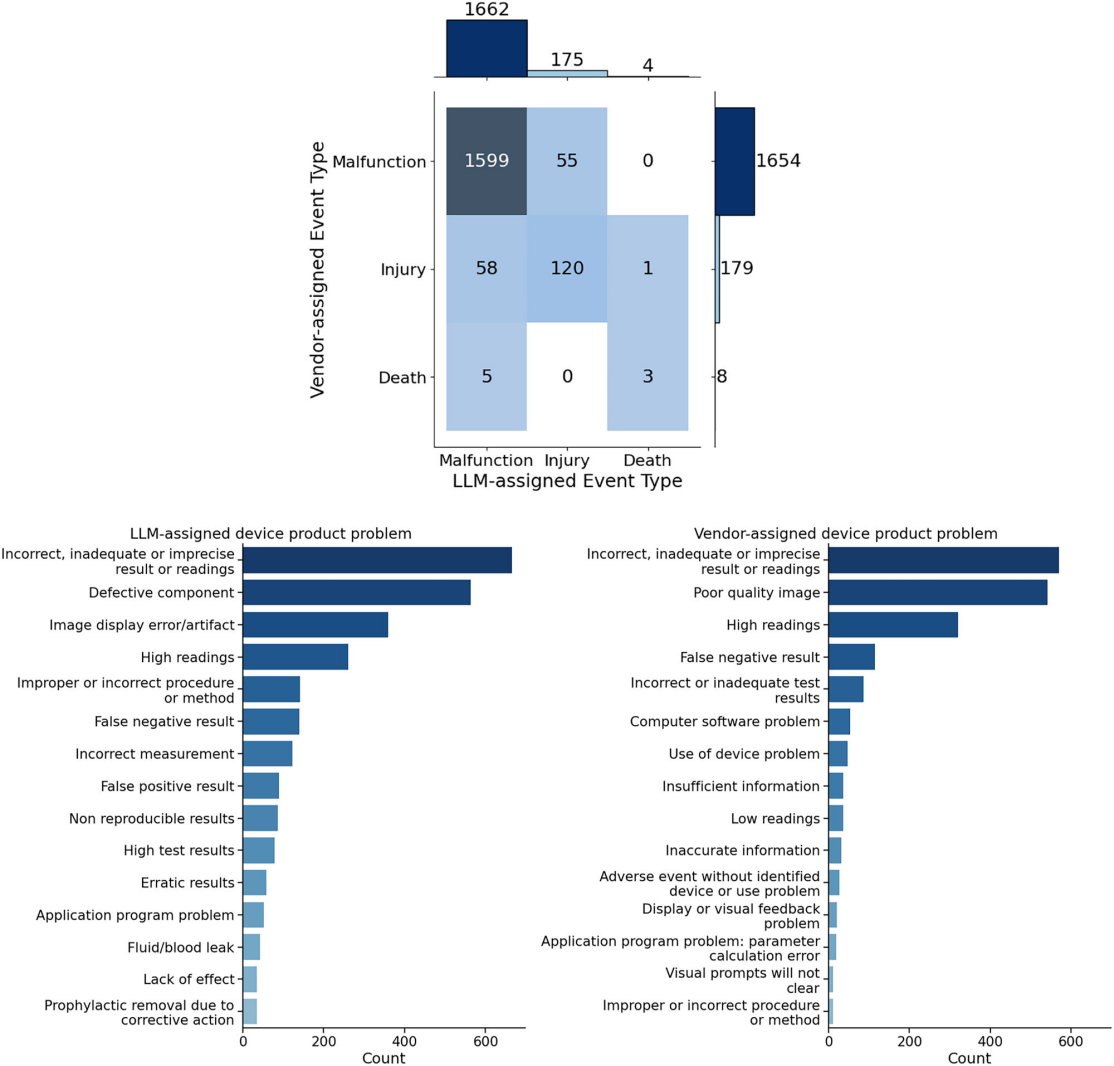
Categorization of medical device reports. The top row compares event types assigned by the LLM pipeline and vendor. The bottom-left and right bar charts compare the device product problem codes assigned by the LLM pipeline and vendor, respectively.

Given the LLM validation results, we compare Event Types assigned by the LLM versus vendor (Fig. 3). The LLM disagreed with the vendors on the Event Type code for 119 of the 1841 MDRs. Although disagreement rates were generally low for event type codes, disagreements involving the “death” label were overrepresented. Given the severity of this event type, we had all four human annotators review all six disagreements involving “death” labels. All human annotators agreed with the LLM on the one MDR that was upgraded from “injury” to “death” (MDR report number 3002808157-2024-00004), which stated “*78-year-old patient with a stroke alert was examined on a magnetom aera system… The patient was aphasic and thus did not understand what was said to her… A head coil was used for the examination… the patient moved her head inside the head coil a lot during examination, without moving other parts of her body. One of the two technicians entered the examination room to ask the patient to stop moving to improve the image quality. When the technician entered, they found vomit all over the examination table… [and the patient] was not breathing… resuscitation of the patient was not successful, and the patient died*.” The report stated that the head coil and “*the positioning of the mri surveillance camera did not make it possible to detect the patient’s discomfort*.” The human annotators also unanimously agreed with the LLM on downgrading from “death” to “malfunction” for three of the five MDRs, many of which explicitly stated in the MDR text that the device was unlikely to have caused or contributed to death. Of the remaining MDRs, most human annotators agreed with the LLM.

In contrast to event type codes, the LLM often disagreed with the vendor on the appropriate device product problem code. Although the most frequent DPP among both LLM-assigned and vendor-assigned codes was “Incorrect, inadequate or imprecise result or readings,” the second-most common DPP was “poor quality image” among vendor-assigned codes and “defective component” for LLM-assigned codes. Thus, per the LLM, the second-most common device product problem reported is one relating to the device hardware rather than software. Investigating the labeling differences, we find that the reason for this shift is that the “poor quality image” code is primarily associated with MDRs for a general-purpose diagnostic ultrasound system called Voluson SWIFT (K230346), in which the MDR texts described how an “*ultrasound probe… [was] having a component malfunction*” that led to an imaging artifact (see e.g. MDR number 8020021-2025-00173). However, the comprehensiveness of the MDR text varies, with some only describing a malfunctioning ultrasound probe and others mentioning the imaging artifacts as well. As such, the LLM tended to select the DPP codes of “defective component” or “defective component” and “image display error/artifact.” Consequently, by having the LLM pipeline suggest DPP codes, it can identify gaps in the alignment between codes assigned by a human coder and the corresponding MDR text, which could either be addressed by modifying the selected codes or the MDR text itself.

### Case study 3: Identifying premarket risk factors

Understanding the association between premarket device characteristics and post-market adverse events is critical for advancing medical device regulation. Factors such as predicate device selection, specific device modifications, and the type of validation studies conducted during the 510(k) clearance process may influence a device’s real-world safety profile. However, establishing these links has traditionally been challenging due to the manual and labor-intensive nature of extracting relevant information from unstructured regulatory documents and the complexities of linking premarket data with post-market surveillance. This case study illustrates how LLMs can be leveraged to overcome these barriers.

Using the LLM pipeline, we extract premarket attributes and link them to categorized post-market MDRs, focusing on hardware, software, and human factor errors (Table 3). Validating the pipeline, primary predicate extractions showed 99% agreement with^6^, and other premarket attributes showed an average agreement rate of 80% with human annotators across 78 randomly sampled devices. For reference, the interrater agreement was similar, with a percent agreement of 86.43% (80.0%, 92.8%).

**Table 3:** Definitions and examples of premarket attributes, as well as their prevalence per LLM extractions

	Attribute	Definition	Example	Prevalence	Accuracy
**Predicate selection**	Primary predicate	The primary predicate of the subject device.	K240062 uses K203115 as its predicate.	N/A	98% (96%, 100%)
**Device validation**	Clinical testing	If the summary mentions any clinical validation studies	K043341 was “test[ed on] samples prospectively collected from patients seen in a rheumatology clinic.”	54%	81% (70%, 93%)
	Human+AI Testing	If the summary mentions assessing the performance of the human device team, beyond that of the device in isolation	K190017 conducted “performance testing using in-vivo volunteer data to assess the precision of LMSv3, inter- and intra- operator variability and the worst-case variability.”	27%	83% (72%, 94%)
**Device difference**	Intended use and clinical applications	If changes in intended use, clinical indications, target conditions, and/or patient populations were mentioned	K192973 significantly differed in “addition of breast tomosynthesis systems.”	30%	70% (57%, 84%)
	Clinical operation and workflow	If changes in workflow or efficiency (e.g., automation, major UI/UX, system integration) were mentioned	K251071 differs in that its “outputs are annotated DICOM files displayed in a DICOM viewer and may optionally be displayed on a web interface,” while “outputs of the predicate device are annotated DICOM files displayed in a DICOM viewer.”	29%	80% (68%, 92%)
	Algorithm/ software features	If major changes in the algorithm/ML model type or new software features were mentioned	K211541 has updated “algorithmic components… to improve detection accuracy for FFDM and to enable processing of DBT.”	66%	76% (64%, 89%)
	Hardware	If changes to physical components, sensors, or form factor to the device itself or its inputs were mentioned	K210611 “comes with significant new and modified hardware,” including the addition of new magnets, coils, and cameras	20%	89% (80%, 98%)
	Body part	If the subject device targets a new body part and/or significantly expands the range of body parts	K240953 targets “Heart, Lungs,” while the predicate device targets “Heart, Bladder.”	16%	83% (72%, 94%)

Prevalence is how often the type of device validation or device difference was mentioned in the device summary.

Accuracy is how often the LLM extraction exactly matched the human annotation, assessed using 78 randomly sampled devices for device validation and device difference attributes (see Methods section for details) and ref. ^6^ for the “primary predicate” attribute.

The LLM pipeline was then scaled up to extract premarket attributes of all AI/ML-enabled medical devices to identify potential associations between premarket factors and post-market MDRs. Given known limitations in the raw MDR and 510(k) data^7^, we emphasize that this analysis is exploratory and is intended to demonstrate what is possible with current LLM capabilities. Using Cox proportional hazard models, we found several premarket factors to be statistically associated with time to first MDR (Table 4). Consistent with prior findings, selecting a predicate device with a history of recalls or MDRs was associated with increased hazard^27^. Hardware changes also showed significant associations with higher hazard rates across most MDR types. Conversely, clinical testing for obtaining premarket approval/clearance was associated with reduced hazard rates.

**Table 4:** Estimated associations between premarket device clearance factors and post-market MDRs

	Any MDR		Hardware MDR		Software MDR		Human MDR	
Variable	HR (95% CI)	*p* val	HR (95% CI)	*p* val	HR (95% CI)	*p* val	HR (95% CI)	*p* val
Primary predicate								
… has MDR	9.19 (4.70, 18.00)	0.00*	7.96 (3.40, 19.00)	0.00*	6.77 (2.90, 16.00)	0.00*	12.28 (4.60, 33.00)	0.00*
… has recall	6.49 (3.40, 12.00)	0.00*	5.60 (2.30, 13.00)	0.00*	5.17 (2.20, 12.00)	0.00*	8.96 (3.20, 25.00)	0.00*
Validation testing								
Clinical testing	0.43 (0.24, 0.75)	0.00*	0.48 (0.24, 0.97)	0.04*	0.39 (0.19, 0.78)	0.01*	0.63 (0.27, 1.50)	0.28
Human + AI team testing	1.31 (0.75, 2.30)	0.35	1.26 (0.63, 2.50)	0.51	1.28 (0.64, 2.60)	0.49	1.37 (0.58, 3.2)	0.47
Device differences								
Intended use	0.83 (0.42, 1.60)	0.6	0.68 (0.29, 1.60)	0.38	0.94 (0.41, 2.10)	0.89	0.62 (0.20, 1.90)	0.41
Hardware	2.54 (1.60, 4.10)	0.00*	2.86 (1.50, 5.30)	0.00*	1.42 (0.75, 2.70)	0.28	2.48 (1.20, 5.30)	0.02*
Software or algorithm	1.52 (0.86, 2.70)	0.15	3.84 (1.50, 10.00)	0.01*	1.16 (0.58, 2.30)	0.67	1.55 (0.61, 4.00)	0.36
Body part	1.43 (0.68, 3.00)	0.34	1.56 (0.63, 3.90)	0.33	1.57 (0.63, 3.90)	0.34	1.86 (0.61, 5.70)	0.28
Operation or workflow	0.99 (0.60, 1.60)	0.98	0.84 (0.45, 1.60)	0.59	1.24 (0.66, 2.30)	0.51	1.24 (0.57, 2.70)	0.59

## Discussion

Given the rapid increase in AI/ML-based medical devices, there is a need for accurate and scalable approaches for surveying the regulatory landscape and tracking the full lifecycle of these devices. This work presents the first wide-ranging validation study of LLM’s ability to assist in this role as an information extractor. Validated against manual and LLM-as-a-judge annotations, we have shown that LLMs can accurately extract critical information from complex, unstructured regulatory documents from both premarket and post-market settings. The extracted information can then be used to support various analyses across the regulatory lifecycle, such as monitoring changes in device validation practices (case study 1), highlighting the most common types of Medical Device Reports (case study 2), and generating hypotheses about the impact of device clearance practices (case study 3). Conducting these case studies previously required months, if not years, to complete. Now, LLMs can complete these analyses within days. This work demonstrates how LLMs have the potential to transform regulatory science by enabling more efficient, accurate, and data-driven analyses of medical device evaluation and post-market surveillance practices.

The LLM pipeline in this work substantially improves on prior works that have used NLP to analyze medical device regulatory documents. Past studies have primarily relied on exact pattern-matching, which limits extractions to simple, well-structured data elements^15,17^, and lacks the reasoning needed for accurate extraction of more complex concepts. Other works have explored black-box NLP methods like using text embeddings to quantify device similarity, but such methods are difficult to interpret and validate^38^. In contrast, LLMs can conduct more sophisticated reasoning and produce auditable reasoning traces, leading to better performance and reliability (see Supplementary Materials for a performance comparison). The FDA is now also exploring the use of LLMs in the regulatory submission process^39^, demonstrating how LLMs may one day be used not only in regulatory science but also in regulatory affairs. Although there is currently no publicly available information on the performance of the FDA’s LLM pipeline, this work demonstrates how LLMs were at least able to conduct related tasks with high accuracy.

We acknowledge a number of limitations in this work, which can be categorized as those relating to the analysis of the data and those relating to the quality of the data itself. Regarding the analysis of the data, we first note that this LLM pipeline demonstrated accuracy rates around 80%, which can be sufficient for many but not necessarily all use cases. For instance, this level of accuracy is often sufficient for conducting downstream statistical analyses of population-level trends, as long as one uses appropriate methods to adjust for the potential extraction errors by the LLM. For instance, methods from the emerging area of Prediction-Powered Inference (PPI) are designed to combine large-scale extractions from AI models with small gold standard datasets to provide unbiased and precise estimates with appropriate uncertainty quantification^40–42^ Second, the validation studies in this work focus on average performance, but performance is likely to vary across device types, years, or submission formats^43,44^, Studying this variation will likely require substantially more human annotations, which we leave to future work^45,46^, For long-term deployment of this LLM pipeline, its performance will need to be monitored^47,48^, perhaps using methods like LLM-as-a-judge for its scalability. Third, the performance of the LLM pipeline may differ if alternative LLMs are used instead. This work focused on the use of GPT-4.1 so we expect similar performance from LLMs with similar reasoning abilities, but performance will likely vary if weaker or more powerful models are used. Finally, we emphasize that LLM analyses, and in fact all computational analyses, must be carefully interpreted with expert judgment. They provide just one piece of evidence, which must be contextualized against other sources of evidence.

This work also highlights current data limitations, as well as potential opportunities. Although this work validated the extraction accuracy of LLMs in medical device regulatory documents and demonstrates what is possible with current LLM capabilities, the underlying data quality still needs to be improved for the LLM-based analyses–or any data analyses–to be robust. In particular, publicly available decision summaries provide an incomplete picture of the device clearance process^49^, as the full regulatory submission is often hundreds of pages. Furthermore, under-reporting and variable quality in the MAUDE database may bias prevalence estimates and hazard ratios^7^. There is also debate over how AI/ML-enabled devices should be defined and whether the FDA’s list of cleared/approved AI/ML-enabled devices is accurate^12^. As such, efforts by the FDA to boost the quality and completeness of publicly available data (see e.g., ref. ^50^) will be critical to enabling robust analysis of regulatory data. This is now a key remaining bottleneck for scaling up regulatory science, given that technologies like LLMs are now able to unlock the information hidden within unstructured documents.

Despite these limitations, our study demonstrates the potential of LLMs to automate monitoring of the regulatory lifecycle amidst an evolving landscape of AI/ML-based medical devices and provide timely, data-driven evidence to support the development of regulatory policies and guidance. Moreover, even though this work has focused on AI/ML-based medical devices, the LLM pipeline can be extended to encompass non-AI/ML-based medical devices as well, to make regulatory data for all device types accessible to everyone.

## Methods

### Dataset

**Fig. 4:**
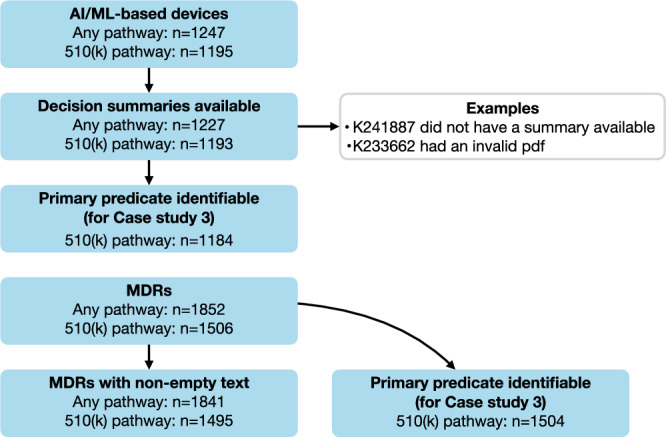
Data preprocessing steps. The data analyzed in this work encompass decision summaries and medical device reports (MDRs) of all FDA-cleared/approved AI/ML-based medical devices up to July 2025.

AI/ML-enabled devices were identified using the list published on the FDA website^1^. Device summaries and MDRs were downloaded via the FDA API. All AI/ML-based devices and their MDRs up to July 2025 were analyzed. The preprocessing pipeline is shown in Fig. 4. A summary of the data is displayed in Table 5.

**Table 5:** Summary of the data analyzed by the LLM pipeline

Data	Summary statistics
Decision summary	*n* = 1227
Number of pages	Mean = 11.3, Min = 3, Max = 77
Medical device report	*n* = 1841
Number of characters	Mean = 776.4, Min = 37, Max = 8636
Number of words	Mean = 120.5, Min = 4, Max = 1334

### LLM pipeline description

The full set of attributes that are extracted by this pipeline are listed in Table 6. The LLM used in the pipeline is GPT-4.1 (with no fine-tuning). Extraction of attributes from device summary PDFs is a two-step process:

1. **Preprocessing device summary PDFs**: Text is extracted from the PDF. In situations where the text is surfaced in an image, text is extracted using the optical character recognition (OCR) software package tesseract^51^.
2. **Information extraction using LLM**: The LLM is asked to extract device validation characteristics, given a prompt containing the device summary text. Prompts are available in the supplement.

**Table 6:** Attributes extracted using the LLM pipeline

Category	Attribute	Type	Definition
Device summary: metadata	Predicates	List of strings	All predicate device numbers are explicitly stated in the device summary
	Primary predicate	String	The primary predicate device number from the list of predicates. In general, there should be only one primary predicate for a device.
Device summary: validation studies	Number of sites	Number	The number of sites used to assess the subject device. If the summary does not provide the exact number but mentions a lower bound, use that instead.
	Multiple-sites	Binary	If the number of sites for the device validation study is two or more
	Prospective	Binary	If the summary mentions that data were collected prospectively to assess the device’s performance
	Clinical testing	Binary	If the summary mentions any clinical device validation studies
	Human + AI testing	Binary	If the summary mentions assessing the performance of the human device team, beyond that of the device in isolation
Device summary: types of differences between the subject device and the primary predicate device	Intended use and clinical applications	Binary	If changes in intended use, clinical indications, target conditions, and/or patient populations were mentioned
	Clinical operation and workflow	Binary	If changes in workflow or efficiency (e.g., automation, major UI/UX, system integration) were mentioned
	Algorithm/ software features	Binary	If major changes in the algorithm/ML model type or new software features were mentioned
	Hardware	Binary	If changes to physical components, sensors, or form factor to the device itself or its inputs were mentioned
	Body part	Binary	If the subject device targets a new body part and/or significantly expands the range of body parts
MDR	Type of reportable event	Categorical	- Death: If the device caused or contributed to the patient’s death. - Injury: If the device caused or contributed to a serious injury of the patient. - Malfunction: If the device is failing to meet performance specifications as intended and/or if neither Death nor Injury are applicable.
	Medical device problem	List of strings	The lowest-level, most detailed code or codes from IMDRF Medical device problem codes Annex A that most accurately describe the device failures or problems observed during the event.

Extraction of attributes from medical device reports from MAUDE also involves a two-step process:

1. **Context engineering:** To ensure the LLM extractions are aligned with regulatory policies and definitions, excerpts from regulatory policies and guidelines are also provided to the LLM. In particular, we include instructions from “FDA 3500A MedWatch Supplement Form Instructions,” FAQs from “medical device reporting for manufacturers,” instructions from the FDA’s MDR coding manual, and the full hierarchy of medical device problem codes.
2. **Information extraction using LLM**: The LLM is asked to assign codes to the MDR given the device name, MDR text (includes “manufacturer narrative” and “event description”), and the engineered regulatory context.

To improve the LLM’s extraction accuracy, all prompts ask the LLM to reason in a step-by-step manner^52^ and provide examples for In-Context Learning^53^. Structured Outputs are also used so that generated outputs follow a consistent schema. To minimize stochasticity, LLM outputs were generated with the temperature set to zero. All prompts are provided in the Supplementary Materials.

### Comparing extractions from pattern-matching versus LLM

We compared extractions from an LLM to those from exact pattern-matching. We re-extracted all the binary and categorical features used in this study by selecting keyphrases corresponding to each attribute based on prior knowledge. The attribute’s value is then determined by whether any of its corresponding keyphrases were present. For instance, the “multi-site” attribute is positive if any of the following keyphrases were present in the decision summary: “clinical sites”, “multiple sites”, “multiple centers”, “multiple hospitals”, “multisite”, “multicenter”, “multi-site”, “multicenter.” We then evaluated the agreement rate between rule-based extractions and human annotations, which is presented below in Table 7. The agreement rates for the LLM pipeline are presented as well for convenience.

**Table 7:** Agreement rates between rule-based extractions and human annotations, versus agreement between LLM pipeline extractions and human annotations

Attribute	Rule-based method	LLM pipeline
Multi-site	72% (62%, 82%)	94% (87%, 100%)
Prospective	76% (67%, 86%)	94% (87%, 100%)
Clinical Testing	50% (38%, 61%)	82% (70%, 93%)
Human + AI testing	77% (67%, 87%)	82% (72%, 93%)
Intended use and clinical applications	69% (58%, 80%)	70% (57%, 83%)
Clinical operation and workflow	68% (58%, 79%)	80% (68%, 91%)
Algorithm/software features	37% (26%, 48%)	76% (64%, 89%)
Hardware	76% (67%, 86%)	89% (80%, 98%)
Body part	88% (80%, 95%)	83% (73%, 94%)
MDR: Event type	72% (63%, 81%)	93% (88%, 98%)

### Human annotation validation

Three datasets were used to validate the performance of the LLM pipeline:

- 130 device summaries were annotated in ref. ^6^ for the following attributes: predicate device numbers, number of sites, multi-site, and prospective.
- 78 device summaries were annotated in this work for device summary attributes in Table 6.
- 89 medical device reports annotated in this work for MDR attributes in Table 6.

The processes for generating annotations in this work are described below.

#### Device summaries

A total of 78 unique device decision summaries were annotated by two data scientists and two regulatory experts. To adjust for the lower prevalence of multisite and prospective studies, decision summaries were selected through stratified random sampling. Each annotator labeled 7 or 8 decision summaries from the three buckets based on the LLM’s label: (i) having an LLM-assigned label of “did conduct prospective validation,” (ii) having an LLM-assigned label of “did conduct a multisite validation study” but no LLM-assigned label of “did conduct prospective validation,” and (iii) having neither of the two labels. Any label that a data scientist annotator was unsure about was adjudicated by a regulatory expert annotator. Of the 78 decision summaries, 11 were dual-annotated by the two regulatory experts to assess interrater reliability. Due to the stratified sampling, note that the agreement rates in Case Study 3 are after weighting the annotated data to reflect the original data distribution.

#### Medical device reports

A total of 90 unique MDRs were annotated by two data scientists and two regulatory experts. Annotators were asked whether they preferred the LLM-assigned or vendor-assigned code. To avoid bias, the annotator was blinded to the origin of each code, and the ordering of the codes was randomized. The MDRs were selected via stratified random sampling, in which 45 were sampled from MDRs where the LLM-assigned and vendor-assigned codes disagreed for Event Type and another 45 were sampled from MDRs where there was disagreement for the Device Product Problem. In addition, all MDRs where either the vendor or LLM assigned “death” as the event type were included for annotation by all four annotators. To assess interrater reliability, a total of 30 MDRs were dual annotated.

### LLM-as-a-judge for MDRs

LLM-as-a-judge was used to judge if LLM-assigned or vendor-assigned codes were more appropriate. LLM-as-a-judge was implemented using GPT-4.1 and Claude Sonnet 4.5. The LLM judges were given the same instructions as those given to human annotators. Similarly, the LLM judges were shown both code assignments but were blinded to the origin of the code and the ordering of the codes was randomized to minimize the chance of position bias.

### Statistical methods

Given human annotations, the accuracy of LLM extractions was defined as an exact match between the human annotation and the LLM extraction. For continuous features, the mean absolute error (MAE) between human and LLM annotations are also reported. Interrater reliability is quantified in terms of percent agreement. Associations between premarket device characteristics and time-to-first MDR in the MAUDE database were quantified using Cox proportional hazards models, incorporating random effects to account for clustering within medical specialty panels. Hazard ratios (HR) with corresponding 95% confidence intervals are reported. All statistical analyses were performed using R 4.3.0.

## Supplementary information

Supplementary Information

## Data Availability

All raw data used for the manuscript is available on the FDA website.

## Code availability

Code for processing the data analyzed in this work and reproducing the results is attached in the supplement and is publicly available at https://github.com/jjfenglab/LLM-FDA-device.
