# Supplementary material for: Scaling medical device regulatory science using large language models
Source: NPJ Digit Med. 2026 Feb 5;9:221. doi: 10.1038/s41746-026-02353-7 (PMC12992660; doi:10.1038/s41746-026-02353-7)
Supplement: Supplementary file 1 — Supplementary Information [file 41746_2026_2353_MOESM1_ESM.pdf]

Supplementary Materials

LLM prompt for extracting validation study characteristics:

You will be shown a parsed text from a device decision summary of a medical device released by the FDA.
Your task is to extract key information about the device's predicate(s) and clinical study characteristics.
Output one flat JSON object with the specified keys and value types.

Here is a text from the parsed device decision summary: {text}

Instructions:

Task 1: Predicate Identification
- "predicates": (List[str]) All predicate device K numbers explicitly stated in the summary, selected from: {potential_predicate_numbers}
- "primary_predicate": (str) The primary predicate K number, chosen from "predicates". There should be one and only one primary predicate for a device. If none can be confidently identified, use an empty string.

Guidelines:
- Notice that not all device numbers mentioned in the summary are predicates of the target device. Please read the text to find the device numbers that are explicitly stated as predicates.

Task 2: Validation Study Characteristics
- "num_sites_reason": (str) Explain how the number of sites (single vs multisite and exact number of sites) was determined, along with a quote. If the exact number of sites is not mentioned, extract any lower bound that is mentioned. If there is no information whatsoever, mention this instead.
- "num_sites": (int) The number of sites used to validate the subject device, either prospectively or retrospectively. If the summary does not provide the exact number but mentions a lower bound, use that instead. Otherwise, if no information is available at all, set to null.
- "is_multisite": (boolean) The number of sites used to validate the subject device, either prospectively or retrospectively, is two or more. False otherwise.
- "is_prospective_reason": (str) Explain the reasoning for whether prospective validation was conducted, along with a quote.

- "is_prospective": (boolean) True if there was data was collected prospectively to validate the device's performance. False otherwise.

Suggestions for analyzing validation studies:
- Look for mentions of clinical studies, validation studies, or performance evaluations
- Search for terms like "prospective", "retrospective", "concurrent", "real-time data collection"
- Count the number of sites/centers/institutions mentioned in clinical evaluation
- If multiple studies are mentioned, focus on the primary clinical validation study

Examples:
- "A prospective study was conducted at the US and Korea" → num_sites: 2, num_sites_reason: "The text did not state the exact number of sites, but it does mention validating across two countries.", is_prospective: true, is_prospective_reason: "The study is explicitly described as prospective"
- "Retrospective analysis of data from 3 hospitals" → num_sites: 3, num_sites_reason: "The text mentions '3 hospitals' used for data analysis", is_prospective: false, is_prospective_reason: "The analysis is described as retrospective"
- "Multi-site validation study across 10 centers" → num_sites: 10, num_sites_reason: "The text states '10 centers' were involved", is_prospective: false, is_prospective_reason: "No indication of prospective data collection, appears to be validation on existing data"

JSON format of structured output:
{
  “predicates”: <list>,
  “primary_predicate”: <str>,
  “num_sites”: <int>,
  “num_sites_reason”: <str>,
  “is_multisite”: <bool>,
  “is_prospective”: <bool>,
  “is_prospective_reason”: <str>,
}

LLM prompt for classifying problem type in Medical Device Reports:

Carefully review the following Medical Device Report (MDR), which must be filed with the FDA.

Medical Device Report Text:
<mdr_text>
Device name: {device_name}
{event_text}
</mdr_text>

Your task is to determine what values to fill out in Form FDA 3500A MedWatch for the entries "Type of Reportable Event" and "Medical Device Problem".

For "Type of Reportable Event", the MDR should be classified into one of the following three categories per FDA 3500A SUPPLEMENT FORM INSTRUCTIONS:
- Death: Check only if the MDR reportable event represents a device-related death.
- Injury: The MDR reportable event represents an adverse event that is life-threatening; results in permanent impairment of a body function or permanent damage to a body structure; or necessitates medical or surgical intervention to preclude permanent impairment of a body function or permanent damage to a body structure.
- Malfunction: Failure of a device to meet its performance specifications or otherwise perform as intended. Performance specifications include all claims made in the labeling for the device. The intended performance of a device refers to the intended use for which the device is labeled or marketed. If neither Death nor Injury are applicable, choose Malfunction.

The FDA guidance document "Medical Device Reporting for Manufacturers" provides the following FAQs to guide the classification of the Event Type:
* What are "MDR Reportable Events"?
  > For manufacturers, "MDR reportable events" are events where the manufacturers become aware of that reasonably suggest that one of their marketed devices may have caused or contributed to a death or serious injury, or has malfunctioned and the malfunction of the device or a similar device that they market would be likely to cause or contribute to a death or serious injury if malfunction were to recur.
* What is meant by "caused or contributed" to a death or serious injury?
  > This means that a death or serious injury was or may have been attributed to a medical device or that a medical device was or may have been a factor in a death or serious injury, including events occurring as a result of [21 CFR 803.3] failure, malfunction, improper or inadequate design, manufacture, labeling, or user error.

* What is device "user error" and why do you want to know about events involving user error?
  > We consider a device "user error" (or "use error") to mean a device-related error or mistake made by the person using the device. The error could be the sole cause of an MDR reportable event, or merely a contributing factor. Such errors often reflect problems with device labeling, the user interface, or other aspects of device design. Thus, FDA believes that these events should be reported in the same way as other adverse events which are caused or contributed to by the device. This is especially important for devices used in non-health care facility settings. If you determine that an event is solely the result of user error with no other performance issue, and there has been no device related death or serious injury, you are not required to submit an MDR report, but you should retain the supporting information in your complaint files.
* What is a "serious injury"?
  > An injury must meet the definition of "serious injury" in 21 CFR 803.3 for an event to be reportable as a serious injury. A "serious injury" is an injury or illness that [21 CFR803.3]: is life threatening, results in permanent impairment of a body function or permanent damage to a body structure, or necessitates medical or surgical intervention to preclude permanent impairment of a body function or permanent damage to a body structure."Permanent" means irreversible impairment or damage to a body structure or function, excluding trivial impairment or damage [21 CFR 803.3]. Note that not all cosmetic damage will be considered trivial. Furthermore, a life-threatening injury meets the definition of serious injury, regardless of whether the threat was "temporary." It should also be noted that a device does not have to malfunction for it to cause or contribute to a serious injury. Even though a device may function properly, it can still cause or contribute to a death or serious injury.

For "Medical Device Problem", assign one or more codes to the following MDR. Instructions from the FDA's MDR Coding Manual:
* The FDA MDR adverse event codes are divided into the following seven categories:
  - "Medical Device Problem": Problems (malfunction, deterioration of function, failure) of medical devices
  - "Medical Device Component": The parts and components which were involved in, or affected by, the medical device adverse event/incident.
  - "Cause Investigation: Type of Investigation": What was investigated and what kind of investigation was conducted to specify the root cause of the adverse event.

- "Cause Investigation: Investigation Findings": The findings in the specific investigation that are the keys to identify the root cause of the event.
- "Cause Investigation: Investigation Conclusion": The conclusion regarding the root cause of the reported event.
- "Health Effects: Clinical Signs and Symptoms or Conditions": The clinical signs and symptoms or conditions of the affected person appearing as a result of the medical device adverse event/incident.
- "Health Effects: Health Impact": The consequences of the medical device adverse event/incident on the person affected.

* Code Structure: Each set of codes is organized in a tree-like hierarchical structure, where higher-level (closer to the root) codes are more generic, while lower-level (leaf) codes are more specific. For instance, "IMDRF:A01" is higher-level because it has fewer digits while "IMDRF:A010101" is the lowest level with 6 digits. A parent code is often divided into multiple distinct and more-specific child codes, each of which can be considered a member of the set of problems or observations described by the parent code. This allows each set of codes to be intuitively organized in a way that accurately represents the relationship between different but similar codes.
* Reporters should code to the lowest level possible; in other words, they should choose the most specific term(s) available in each category to describe the event or investigation. Reporters may choose more than one code from each set when filing their report, but there is no need to choose both a parent code and one of its children; by definition, the child code is a member or type of the problem or observation represented by its parent, so the child code alone is sufficient.

Your task is to only focus on the category "Medical Device Problem". Carefully go through the following list of medical device problem codes from the FDA and reason through how the codes should be assigned, step-by-step. First consider the highest level codes (IMDRF:AXX with two digits) and then select a more detailed version.
The list of FDA codes are:
Patient Device Interaction Problem,IMDRF:A01
Biocompatibility,IMDRF:A0101
Device Appears to Trigger Rejection,IMDRF:A010102
...
Prophylactic removal due to corrective action,IMDRF:A2501
Insufficient Information,IMDRF:A26
Appropriate Term/Code Not Available,IMDRF:A27

Provide your output in the following JSON format, where "event_type" provides is the assigned label for "Type of Reportable Event" and the

"fda_device_problem_codes" is a list of strings with one or more relevant CDRH Preferred Terms (not the IMDRF codes).

Example JSON: {
    "reasoning": "<DESCRIBE YOUR CHAIN OF THOUGHT HERE>",
    "event_type": "Death", # options: "Death" or "Injury" or "Malfunction"
    "fda_device_problem_codes": ["Lack of Effect"]}

LLM prompt for classifying pre-market characteristics:

You will be shown a parsed text from a 510(k) summary of a medical device released by the FDA.
Your task is to extract key information about the device, its predicate(s), differences the subject and predicate devices, and validation methods.
Output one flat JSON object with the specified keys and value types.

Here is a text from the parsed 510(k) summary: {text}

Instructions:

Task 1: Predicate Identification
- "predicates": (List[str]) All predicate device K numbers explicitly stated in the summary, selected from: {potential_predicate_numbers}
- "primary_predicate": (str) The primary predicate K number, chosen from "predicates". There should be one and only one primary predicate for a device. If none can be confidently identified, use an empty string.

Guidelines:
- Notice that not all device numbers mentioned in the summary are predicates of the target device. Please read the text to find the device numbers that are explicitly stated as predicates.

Task 2: Differences between Subject and Predicate Devices (all boolean; True only if a clear difference is described)
- "intended_use_and_clinical_applications": Changes in intended use, clinical indications, target conditions, or patient populations.
- "operational_and_workflow_change": Major change in workflow or efficiency (e.g., automation, major UI/UX, system integration).
- "algorithm_or_software_feature_changes": Clear, categorical changes in algorithm/ML model type (e.g., non-AI/ML → AI/ML, rule-based → neural network = True), changes in the algorithm's input/output, major new software features, and/or significant UI/UX changes. A device that

only retrained the same model and nothing else would be False. Vague statements that do not describe substantial differences (e.g., both devices use AI) are also False..
- "hardware_changes": Major, clearly described changes to physical components, sensors, or form factor to the device itself or its inputs.
- "body_part_changes": True if subject targets a body part that is not covered by predicate device or if subject device significantly expands the range of body parts compared to predicate. This is False if the subject device only covers a subset of the body parts covered by the predicate device.

Task 3: Validation Methods (all boolean)
- "human_device_team_testing": True if the performance of the device was assessed for the human+device team, beyond just the performance of the device in isolation. This answers questions like "Did the intended user working with the new device perform the same or better than the operator alone or with a different device?" Examples include: Multi-reader multi-case studies that compare how often readers correctly diagnose a disease from medical images when they are or are not assisted by the medical device.
- "has_clinical_testing": True if the summary mentions any clinical validation (prospective, retrospective, etc.). False if the summary explicitly states that clinical testing is not required or did not mention any clinical testing.

Guidelines:
- Mark a field as true only if the summary clearly describes the information. Do not infer from vague or generic statements.
- If information is not mentioned, mark as false (or use an empty list/string for predicates).
- Focus on the comparison between the subject device and its primary predicate in the Substantial Equivalence section.

JSON format of structured output:
{
  “predicates”: <list>,
  “primary_predicate”: <str>,
  “intended_use_and_clinical_applications”: <bool>,
  “operational_and_workflow_change”: <bool>,
  “algorithm_or_software_feature_changes”: <bool>,
  “hardware_changes”: <bool>,
  “body_part_changes”: <bool>,
  “human_device_team_testing”: <bool>,
  “has_clinical_testing”: <bool>

}

LLM-as-a-Judge Prompt:

```
You are validating categorizations assigned to Medical Device Reports
(MDRs) that are to be submitted to the FDA.

Medical Device Report Text:
<mdr_text>
Device name: {device_name}
{event_text}
</mdr_text>

Review the following instructions from the FDA for selecting values
for "Type of Reportable Event" and "Medical Device Problem" in Form
FDA 3500A MedWatch.

For "Type of Reportable Event", the MDR should be classified into one
of the following three categories per FDA 3500A SUPPLEMENT FORM
INSTRUCTIONS:
- Death: Check only if the MDR reportable event represents a device-
related death.
- Injury: The MDR reportable event represents an adverse event that is
life-threatening; results in permanent impairment of a body function
or permanent damage to a body structure; or necessitates medical or
surgical intervention to preclude permanent impairment of a body
function or permanent damage to a body structure.
- Malfunction: Failure of a device to meet its performance
specifications or otherwise perform as intended. Performance
specifications include all claims made in the labeling for the device.
The intended performance of a device refers to the intended use for
which the device is labeled or marketed. If neither Death nor Injury
are applicable, choose Malfunction.

The FDA guidance document "Medical Device Reporting for Manufacturers"
provides the following FAQs to guide the classification of the Event
Type:
* What are "MDR Reportable Events"?
  > For manufacturers, "MDR reportable events" are events where the
manufacturers become aware of that reasonably suggest that one of
their marketed devices may have caused or contributed to a death or
serious injury, or has malfunctioned and the malfunction of the device
or a similar device that they market would be likely to cause or
contribute to a death or serious injury if malfunction were to recur.
* What is meant by "caused or contributed" to a death or serious
injury?
```

  > This means that a death or serious injury was or may have been attributed to a medical device or that a medical device was or may have been a factor in a death or serious injury, including events occurring as a result of [21 CFR 803.3] failure, malfunction, improper or inadequate design, manufacture, labeling, or user error.
* What is device "user error" and why do you want to know about events involving user error?
  > We consider a device "user error" (or "use error") to mean a device-related error or mistake made by the person using the device. The error could be the sole cause of an MDR reportable event, or merely a contributing factor. Such errors often reflect problems with device labeling, the user interface, or other aspects of device design. Thus, FDA believes that these events should be reported in the same way as other adverse events which are caused or contributed to by the device. This is especially important for devices used in non-health care facility settings. If you determine that an event is solely the result of user error with no other performance issue, and there has been no device related death or serious injury, you are not required to submit an MDR report, but you should retain the supporting information in your complaint files.
* What is a "serious injury"?
  > An injury must meet the definition of "serious injury" in 21 CFR 803.3 for an event to be reportable as a serious injury. A "serious injury" is an injury or illness that [21 CFR803.3]: is life threatening, results in permanent impairment of a body function or permanent damage to a body structure, or necessitates medical or surgical intervention to preclude permanent impairment of a body function or permanent damage to a body structure."Permanent" means irreversible impairment or damage to a body structure or function, excluding trivial impairment or damage [21 CFR 803.3]. Note that not all cosmetic damage will be considered trivial. Furthermore, a life-threatening injury meets the definition of serious injury, regardless of whether the threat was "temporary." It should also be noted that a device does not have to malfunction for it to cause or contribute to a serious injury. ven though a device may function properly, it can still cause or contribute to a death or serious injury.

Here are the proposals by two coders for "Type of Reportable Event":
Option 1: {gen_event_types[rand_idxs[0]]}
Option 2: {gen_event_types[rand_idxs[1]]}

Now review the following instructions from the FDA for selecting values for "Medical Device Problem" per the FDA's MDR Coding Manual:
* The FDA MDR adverse event codes are divided into the following seven categories:

- "Medical Device Problem": Problems (malfunction, deterioration of function, failure) of medical devices
  - "Medical Device Component": The parts and components which were involved in, or affected by, the medical device adverse event/incident.
  - "Cause Investigation: Type of Investigation": What was investigated and what kind of investigation was conducted to specify the root cause of the adverse event.
  - "Cause Investigation: Investigation Findings": The findings in the specific investigation that are the keys to identify the root cause of the event.
  - "Cause Investigation: Investigation Conclusion": The conclusion regarding the root cause of the reported event.
  - "Health Effects: Clinical Signs and Symptoms or Conditions": The clinical signs and symptoms or conditions of the affected person appearing as a result of the medical device adverse event/incident.
  - "Health Effects: Health Impact": The consequences of the medical device adverse event/incident on the person affected.
* Code Structure: Each set of codes is organized in a tree-like hierarchical structure, where higher-level (closer to the root) codes are more generic, while lower-level (leaf) codes are more specific. A parent code is often divided into multiple distinct and more-specific child codes, each of which can be considered a member of the set of problems or observations described by the parent code. This allows each set of codes to be intuitively organized in a way that accurately represents the relationship between different but similar codes.
* Reporters should code to the lowest level possible; in other words, they should choose the most specific term(s) available in each category to describe the event or investigation. Reporters may choose more than one code from each set when filing their report, but there is no need to choose both a parent code and one of its children; by definition, the child code is a member or type of the problem or observation represented by its parent, so the child code alone is sufficient.

Here are the proposals by two coders for the specific category of "Medical Device Problem":
Option 1: {gen_codes[rand_idxs[0]]}
Option 2: {gen_codes[rand_idxs[1]]}

Based on the information above, which assigned values are more appropriate for "Type of Reportable Event" and "Medical Device Problem"? Answer 1 if option 1 is better. Answer 2 if option 2 is better. Answer 0 if both options are equally good (or the two proposed

options are the same) and it should be considered a tie. Assign ties minimally.

Please respond with a JSON object:

```
{
    "event_type":  {
        "judge_reasoning": <PROVIDE YOUR REASONING HERE>,
        "judge_result": <1 or 2 or 0>
    },
    "device_problem_codes": {
        "judge_reasoning": <PROVIDE YOUR REASONING HERE>,
        "judge_result": <1 or 2 or 0>
    }
}
```
